# Peripheral lymphocyte count as a surrogate marker of immune checkpoint inhibitor therapy outcomes in patients with non-small-cell lung cancer

**DOI:** 10.1038/s41598-021-04630-9

**Published:** 2022-01-12

**Authors:** Ye Jin Lee, Young Sik Park, Hyun Woo Lee, Tae Yoen Park, Jung Kyu Lee, Eun Young Heo

**Affiliations:** 1 Division of Pulmonary and Critical Care Medicine, Department of Internal Medicine, Seoul National University Hospital, 101, Daehak-ro, Jongno-gu, Seoul, Republic of Korea; 2 Division of Pulmonary and Critical Care Medicine, Department of Internal Medicine, Seoul Metropolitan Government-Seoul National University Boramae Medical Center, Boramae-gil, 42, Dongjak-Gu, Seoul, Korea

**Keywords:** Cancer, Immunology

## Abstract

Degree of expression of programmed death-ligand 1 (PD-L1) is related with Immune check point inhibitors (ICIs) response but it needs sufficient tumor tissue. There is unmet need for easily accessible and prognostic peripheral blood (PB) biomarkers. We investigated the application of serum peripheral lymphocyte count (PLC) as a predictive PB biomarker for ICI response in patients with NSCLC. We conducted a retrospective study and reviewed the patients with NSCLC who were treated with ICIs from April 1, 2016, to March 31, 2019. The PLC before and after 1 month of immunotherapy was collected. We evaluated the association between PLC and progression-free survival (PFS), overall survival (OS) and adverse events. A total of 231 patients were treated with ICIs for NSCLC. The median follow-up period was 4.7 months and the disease progressed in 138 patients (59.7%). Compared with the lowest quartile (Q1: the lowest 25%), the highest quartile (Q4: the highest 25%) of post-treatment PLC showed a significantly higher PFS (HR 0.28, 95% CI 0.16–0.52) and OS (HR 0.35, 95% CI 0.19–0.65) in the adjusted model. An association between adverse events and PLC was not observed. We revealed that an increased pre- and post-treatment PLC was associated with favorable PFS and OS with NSCLC patients treated with ICIs. PLC could be a helpful for ICI responses in NSCLC.

## Introduction

The introduction of immune checkpoint inhibitors (ICIs), programmed cell death receptor (PD-1) inhibitors (nivolumab or pembrolizumab), and programmed death-ligand 1 inhibitors (PD-L1) inhibitors (atezolizumab) for the standard treatment of advanced non-small-cell lung cancer (NSCLC), has improved survival rates. However, only limited patients receiving ICIs are benefitted^1,2^, because predictive markers in the complexity of the tumor microenvironment is challenging and often requires invasive procedures. PD-L1 expression measured by immunohistochemistry (IHC) has been used as a biomarker in initial clinical trials of PD-1 and PD-L1 inhibitors^3,4^. However, it is ineffective as a single predictive biomarker for ICI treatment; various PD-L1 expression assays with different antibodies, platforms, and cutoffs have been studied, and their outcomes vary from one clinical trial to another. In addition, the heterogeneous expression of PD-L1 in tumors is another limiting factor.

Currently, tumor mutational burden (TMB) is emerging biomarker used in serial CheckMate trials^5^. However, using TMB as a biomarker is also challenging because of the long turnaround time, qualified tissue samples required for accurate analysis, and significant cost involved for the interpretation of next-generation sequencing data used to measure the number of somatic mutations^6^. Tumor-infiltrating T lymphocytes (TILs) are also considered as biomarkers for tumor-immune system interactions; the higher the TIL concentration, the better the effects of ICIs^7,8^. Shortcomings of the TIL-based prognostic biomarker include lack of quantitative IHC approaches, poor inter-observer reproducibility, and time-consuming procedures for the measurement of TIL.

Therefore, there is unmet need for another easy accessible, cheap, and non-invasive predictive markers are required for identifying patients who will benefit from ICIs. Several studies have examined peripheral blood (PB) biomarkers owing to their easy accessibility and low costs^9^. The neutrophil-to-lymphocyte ratio (NLR) is a widely studied blood-based biomarker used for various types of tumors treated with ICIs^10^. Previous studies have shown that a high NLR is associated with poor outcomes in lung cancer^11,12^. Nevertheless, it includes two independent biological factors; neutrophilia and lymphopenia. High NLR has also been evaluated as prognostic factor in acute pancreatitis and cardiac events^13,14^. This finding could be explained by fluctuation neutrophil count which is easily affected by bacterial infection, ischemia, or autoimmune disease and is relatively more frequent than lymphocyte^15^. In addition, several researchers suggested that prognostic value of high NLR might be due to lymphopenia rather than neurtrophilia^16–18^. Therefore, based on these observations, we evaluated the prognostic value of PLC to clinical outcomes in patients with lung cancer. However, the association between peripheral lymphocyte count (PLC) and ICIs has not yet been established. Elevated levels of tumor-infiltrating lymphocytes are considered to be associated with a better prognosis^19^, and decreasing levels of tumor-infiltrating lymphocytes are associated with a poor prognosis in lung cancer^20^. Lee et al., the amount of tumor infiltrating lymphocytes (TILs) was associated with PLC in patients with breast cancer^21^. We hypothesized that PLC might reflect ICI outcomes because the mechanism of ICIs was dependent on the activities of T lymphocytes^22^.Therefore, PLC may be a biomarker for determining ICI responses in NSCLC. In this study, we investigated the application of serum peripheral lymphocyte count (PLC) as a predictive PB biomarker for ICI response in patients with NSCLC.

## Results

### Patient characteristics

A total of 270 patients were diagnosed with lung cancer during the study period at SNUH. Among them, 4 were diagnosed with small cell lung cancer (SCLC), 26 did not undergo PD-L1 IHC assay, and 9 did not have PLC data. Therefore, 39 patients were excluded; 231 patients were finally included, and their data were analyzed in this study. Baseline characteristics of enrolled patients are described in Table 1. Their median age was 66 years (37–86), and 181 (78.5%) patients were men. Most of the patients had good performance status, and 183 (79.6%) were ever-smokers. Twenty-four patients (9.9%) had activating EGFR mutations. Forty-nine patients (21.2%) were treated with ICIs, although they had negative PD-L1 expression. ICI was used as the first- or second-line of therapy in about half of the patients. Most patients were treated with nivolumab (75.3%), and the objective response was observed in 70 (30.3%) patients. During the median observation time, from the first ICI prescription, of 6.7 months (the longest was 45.7 months), 138 (59.7%) cases of progression were observed. The overall median PFS was 83 days and the median OS was 142 days. The median pre- and post-treatment PLCs were 1525.9 cells/µL (1041.9–2070) and 1676.7 cells/µL (1178.6–2190), respectively.

**Table 1:** Baseline characteristics of study patients.

Characteristics	Total (N = 231)
**Age, median (range)**	66 [37–86]
< 65	108 (46.8%)
65–74	76 (32.9%)
≥ 75	47 (20.4%)
**Sex (%)**	
Male	186 (78.5%)
**ECOG (%)**	
0	40 (17.2%)
1	132 (56.9%)
≥ 2	60 (24.8%)
Smoking (ever) (%)	183(79.6%)
**Stage**	
IV	231 (100%)
EGFR activating mutation (%)	24 (9.9%)
**Tumor histologic type (%)**	
Adenocarcinoma	125 (54.1%)
Squamous	56 (24.2%)
Other NSCLC	50 (21.6%)
**PD-L1 expression level (%)**	
≥ 1%	182 (78.8%)
**Line of ICIs used (%)**	
1st	40 (17.3%)
2nd	73 (31.6%)
3rd or more	118 (51.1%)
**Type of ICIs (%)**	
Nivolumab	174 (75.3%)
Pembrolizumab	54 (23.4%)
Atezolizumab	3 (1.3%)
Objective response (OR) (%)	70 (30.3%)
Progression (%)	138 (59.7%)
**PLC, /µL**	
Pre-treatment, median [IQR]	1525.9 [1041.9–2070]
Post-treatment, median [IQR]	1676.7 [1178.6–2190]

OR, partial response and complete response; PLC, peripheral lymphocyte count.

### Treatment outcomes

**Figure 1:**
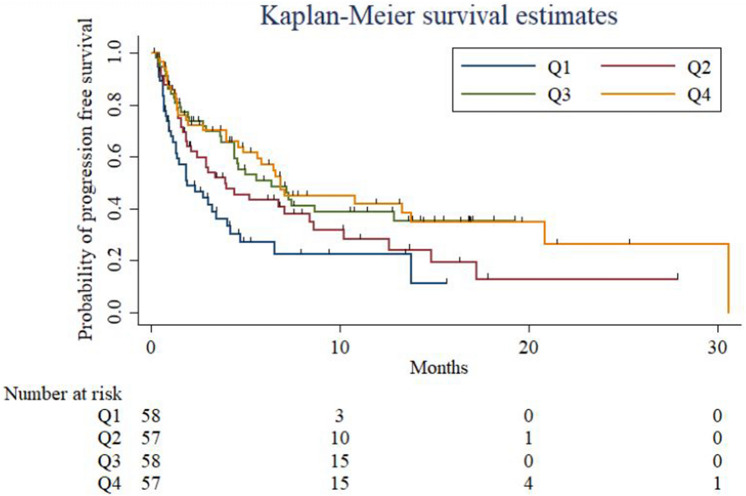
Kaplan–Meier curves showing progression-free survival (PFS) stratified by quartiles of post-treatment PLC.

We found that an increased pre-treatment PLC correlated with slower lung cancer progression and longer PFS after adjusting for age, sex, smoking status, ECOG status, tumor histology, EGFR mutation, and PDL-1 expression (hazard ratio: 0.99, 95% confidence interval: 0.99–1.00 (Supplementary Table 1). Pre- and post-treatment PLCs were divided into quartile groups (Q1–4, Q1 = lowest quartile group of PLC; Q4 = highest quartile group of PLC) for multivariate analysis. PFS was significantly higher in the Q3–4 groups than in the Q1 group for pre-treatment PLC, calculated after adjusting for the factors mentioned above (Table 2, Supplementary Figure S1). The same result was observed for post-treatment PLC, where the Q3–4 groups had a significantly lower progression rate than the reference group, Q1 (Table 3, Fig. 1). Progression decreased for both pre- and post-treatment PLCs across quartile categories (p [for the trend] = 0.006 and 0.001 for pre- and post-treatment PLCs, respectively). Regardless of the pre- and post-treatment PLC values, patients aged > 65 years and ever-smokers were associated with a longer PFS. Besides, patients with EGFR mutations, poor ECOG performance status ≥ 2, and PD-L1 expression levels of at least 1% showed higher progression rates than those were not.

**Table 2:** Multivariate Cox proportional hazard regression analysis for progression-free survival (PFS) and overall survival (OS) according to pre-treatment peripheral lymphocyte count.

	PFS		OS	
	aHR (95% CI)	*P*	aHR (95% CI)	*P*
**Age, years**				
< 65	1 (reference)		1 (reference)	
65–74	0.57 (0.35, 0.94)	0.03	0.71 (0.42, 1.19)	0.19
≥ 75	0.56 (0.32, 0.97)	0.04	0.7 (0.38, 1.28)	0.25
**Sex**				
Male	1 (reference)		1 (reference)	0.58
Female	1.14 (0.68, 1.91)	0.41	1.1 (0.63, 1.89)	
**Smoking status**				0.75
Never	1 (reference)		1 (reference)	
Ever	0.59 (0.35, 1.02)	0.04	0.85 (0.47, 1.52)	
**ECOG (ref 0)**				
0	1 (reference)		1 (reference)	
1	1.54 (0.85, 2.81)	0.18	1.93 (0.96, 3.91)	0.12
≥ 2	2.37 (1.25, 4.51)	0.01	3.61 (1.78, 7.32)	0.001
**Histology**				
Adenocarinoma	1 (reference)		1 (reference)	
Squamous cell carcinoma	1.14 (0.59, 2.20)	0.63	1.00 (0.49, 2.03)	0.86
Other NSCLC	1.41 (0.83, 2.38)	0.32	2.02 (1.19, 3.42)	0.01
EGFR activating mutation	3.24 (1.80, 5.82)	< 0.001	1.90 (0.99, 3.66)	0.10
PD-L1 expression (≥ 1%)	0.43 (0.27, 0.69)	0.001	0.47 (0.28, 0.77)	0.002
**Pre-treatment PLC** **Quartile [25th-75th]**				
1 [144.2–1041.9]	1 (reference)		1 (reference)	
2 [1059.1–1524.4]	0.68 (0.40, 1.17)	0.26	0.53 (0.3, 0.93)	0.004
3 [1527.5–2070]	0.37 (0.20, 0.70)	0.004	0.25 (0.12, 0.51)	< 0.001
4 [2081.3–6241]	0.40 (0.23, 0.71)	0.002	0.36 (0.2, 0.66)	0.002
*P* for trend	0.006		0.035	

PFS, progression-free survival; OS, overall survival; aHR, adjusted hazard ratio; CI, confidence interval; PLC, peripheral lymphocyte count.

**Table 3:** Multivariate Cox proportional hazard regression analysis for progression-free survival (PFS) and overall survival (OS) according to post-treatment peripheral lymphocyte count.

	PFS		OS	
	aHR (95% CI)	*p*	aHR (95% CI)	*p*
**Age, year**				
< 65	1 (reference)		1 (reference)	
65–74	0.49 (0.29, 0.81)	0.006	0.67 (0.39, 1.12)	0.10
≥ 75	0.43 (0.24, 0.75)	0.004	0.51 (0.28, 0.93)	0.02
**Sex**				
Male	1 (reference)		1 (reference)	
Female	0.93 (0.54, 1.57)	0.87	0.94 (0.54, 1.64)	0.84
**Smoking status**	0.49 (0.28, 0.85)			
Never	1 (reference)		1 (reference)	
Ever	0.49 (0.28, 0.85)	0.006	0.65 (0.36, 1.19)	0.2
**ECOG (ref 0)**				
0	1 (reference)		1 (reference)	
1	2.04 (1.09, 3.78)	0.02	2.56 (1.24, 5.26)	0.02
≥ 2	3.00 (1.53, 5.92)	0.001	4.37 (2.11, 9.08)	< 0.001
**Histology**				
Adenocarcinoma	1 (reference)		1 (reference)	
Sqaumous cell carcinoma	1.23 (0.64, 2.35)	0.54	1.16 (0.57, 2.37)	0.57
Other NSCLC	1.52 (0.90, 2.56)	0.18	2 (1.18, 3.40)	0.01
EGFR activating mutation	2.78 (1.54, 5.01)	0.001	1.86 (0.97, 3.61)	0.07
PD-L1 expression (≥ 1%)	0.48 (0.31, 0.78)	0.004	0.55 (0.34, 0.9)	0.01
**Post-treatment PLC,** **Quartile [min–max]**				
1 [208.6–1178.6]	1 (reference)		1 (reference)	
2 [1181–1676.7]	0.61 (0.35, 1.06)	0.082	0.59 (0.32, 1.06)	0.09
3 [1686.7–2180]	0.40 (0.22, 0.73)	0.005	0.29 (0.15, 0.57)	< 0.001
4 [2186.9–5141.7]	0.28 (0.16, 0.52)	< 0.001	0.35 (0.19, 0.65)	< 0.001
*P* for trend	0.001		0.005	

PFS, progression-free survival; OS, overall survival; aHR, adjusted hazard ratio; CI, confidence interval; PLC, peripheral lymphocyte count.

**Figure 2:**
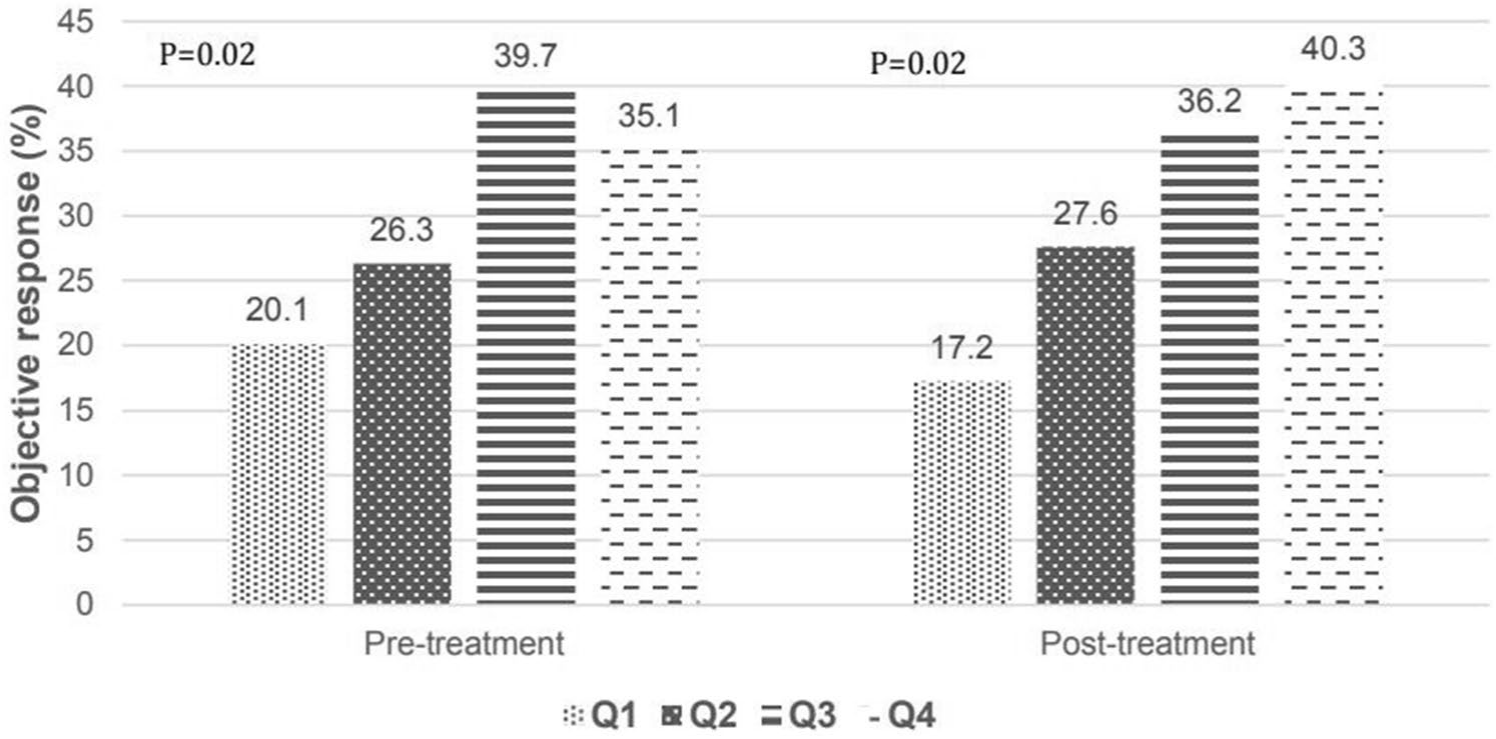
Tumor response rate according to pre- and post-treatment PLC.

We also assessed the effect of PLC on the OS of patients. Pre-treatment PLCs were significantly associated with favorable outcomes in the Q2–4 groups compared with in the Q1 group (Table 2). Post-treatment PLCs exhibited better OS in the Q3–4 groups than in the Q1 group (Table 3). Similar to PFS data, survival increased across both pre- and post-treatment PLC quartile groups (p [for the trend] = 0.035 and 0.005 for pre- and post-treatment PLC, respectively) (Supplementary Figure S2). Patients with poor performance status (ECOG ≥ 2), NSCLC—not otherwise specified, and a PD-L1 expression of at least 1% were associated with poor OS in the multivariate analysis model in both pre- and post-treatment PLC quartile groups, regardless of adjustment. The objective response rate (ORR) increased with the post-treatment PLC level. The ORR was 40.3% in the post-treatment PLC Q4 group, which was more than twice that of the Q1 group (Fig. 2).

Sixty-one patients had irAEs; pneumonitis was the most frequently observed adverse consequence. The frequency and distribution of serious irAEs, fatal or leading to discontinuation of drugs, were similar between both pre- and post-treatment PLC quartile groups (Table 4 and Supplementary Table 2).

**Table 4:** Treatment related adverse events according to posttreatment PLC quartile groups.

Posttreatment PLC	Quart1 (n = 58)	Quart2 (n = 58)	Quart3 (n = 58)	Quart4 (n = 57)	*P*
Adverse event (n = 61)	11 (19.0%)	15 (24.6%)	20 (31.6%)	19(32.1%)	0.30
Pneumonitis	6 (54.6%)	3 (20.0%)	6 (33.3%)	1 (5.3%)	0.43
Skin lesion	1 (9.1%)	2 (13.3%)	4 (20.0%)	3 (15.7%)	
Hepatitis	1 (9.1%)	2 (13.3%)	2(10.0%)	2 (10.5%)	
TFT abnormality	0 (0.0%)	1 (6.7%)	3 (15.0%)	2 (10.5%)	
Arthritis	0 (0.0%)	0 (0.0%)	0 (0.0%)	3 (15.7%)	
Fever	0 (0.0%)	0 (0.0%)	1 (5.0%)	2 (10.5%)	
Anorexia	0 (0.0%)	2 (13.3%)	1 (5.0%)	1 (5.3%)	
Others	3 (27.3%)	5 (33.3%)	3 (15.0%)	5 (26.3%)	
Serious adverse event	6 (10.3%)	5 (8.6%)	7 (12.1%)	3 (534%)	0.67

## Discussion

This study is the first to identify the possibility of predictive marker of PLC for the response of ICIs in NSCLC patients. As expected, an elevated PLC after ICI treatment was associated with prolonged PFS and OS in patients with NSCLC. The PLC level was not related with type of adverse event or prevalence.

A low pre-treatment lymphocyte count tended to be associated with poor prognostic factor for overall survival, relapse, or metastasis in solid tumor^23,24^. The underlying mechanism of pretreatment lymphopenia has not been fully clarified, but this may be attributed to increased apoptosis of TILs as well as circulating lymphocytes or altered lymphocyte homeostasis by over-production of immunosuppressive cytokines like TGF- β or IL-10^25–27^. High-density engagement of TILs, including effector T cells and NK cells, stimulated by tumor-associated antigens, is important for achieving a suitable response to immunotherapy^28^. Zhu et al. showed that melanoma cell with high amounts of Fas-ligand, which then induces apoptosis of tumor-specific CD8 + TILs and their progressive depletion from the periphery, and this is the mechanism resistance to cancer immunotherapy^29^. As we mentioned earlier, the amount of CD8 + TILs was associated with PLC in patients with breast cancer^21^. In melanoma patients with tumor-reactive and specific lymphocytes, PD-1 positive CD8 + lymphocytes were enriched in the peripheral blood^30^. Based on these results, low pretreatment PLC could mean decreased TIL levels in the tumor microenvironment and providing good environment for the tumor cell growth. This may attribute to patients with lowest pretreatment PLC (Q1) having a poor outcome in our study. Although the cut off criteria are slightly different for each study, patients with pre-treatment lymphocyte count less than 1000/mm^3^ or 1500/mm^3^ showed a poor prognosis than those patients who had normal or high levels of lymphocyte, which is consistent considering that the number of pre-treatment PLC in the lowest quartile (Q1), the group with worst prognosis in our study, was less than 1041.9/mm^3^. Pre-treatment blood sampling was usually performed 1 week before the treatment. Most previous studies of NLR and ours used the blood sample drawn 1 week before the treatment to count neutrophils or lymphocytes. In practice, post-treatment blood sampling is performed in between the cycles of immunotherapy. The interval of each treatment cycle can vary according to the types of ICIs. In our study, post-treatment blood sample was taken 3–4 weeks after the first cycle of immunotherapy. Therefore, if we observed the increase of PLC after the first cycle of immunotherapy, we could think that we found responders within 1 month of treatment. In immunotherapy, pseudoprogression is a rare but new pattern of response, which transient immune-cell infiltrate in the tumor bed leading to an artificial increase of the tumor burden. Although more evidence is needed, an increase of PLC in the early phase of immunotherapy should indicate a further continuing treatment and observation instead of discontinuing the treatment.

In multivariate analysis, increased post- treatment PLC, second highest Q3 and highest Q4 has been identified as a good prognostic factor. This may be attributed to the release of neoantigen into peripheral blood, post tumor cell death^31^. This was coherent with results from previous studies that reported that ICIs used in combination with radiotherapy were more effective than ICIs alone^32^. Radiotherapy shows an additive effect with ICIs; the increased release of neoantigens after tumor lysis due to radiotherapy stimulates systemic anti-tumor immunity^33^. Therefore, if patients had a good response to ICIs, peripheral activated effector T-cell expansion may occur due to the emission of neoantigens after tumor cell lysis; this may be reflected as lymphocytosis after treatment. Therefore, lymphocytosis after immunotherapy indirectly refers to the successful treatment of NSCLC.

Our study showed that old people responded better to immunotherapy. This result contradicted results from the CheckMate 227 trial^5^. This may be associated with the difference in the number of patients > 75 years in the two studies; 13 (9%) patients were > 75 years in the CheckMate study, while 37 (15%) patients were > 75 years in ours. On the other side, a recent systematic review showed that elderly patients achieved better outcomes than younger patients, although; this study did not focus exclusively on NSCLC^34^.

Our study also showed that ever smokers had a significantly lower progression rate than never smokers. This finding was consistent with two previous studies, one that showed the effect of nivolumab in advanced NSCLC^35^ and another being a meta-analysis^36^. This might be due to the high TMB in ever smokers compared with never smokers among patients with lung cancer^37^. In addition, our study demonstrated that patients with EGFR mutations had a more rapid progression rate those without. This result is consistent with previous clinical and experimental evidence^38^.

We did not find an association between irAEs and lymphocytosis both pre- and post-treatment. The association between PLC and irAEs remains controversial. Diehl et al. showed that patients with a PLC > 2000 cells/µL at baseline were associated with an increased risk of irAEs. Kamran et al. found that lymphopenia is a predictor for irAEs^39^. Therefore, we were unable to identify if lymphocytosis or lymphopenia was associated with irAEs.

This study has some limitations. First, the timing of blood samples varied for each researches and has not been defined. However, the timing of sample collection within 4 weeks in our study is appropriate, considering T-cell (especially, CD8 + T-cell) which is known to significant in response evaluation, is measured highest at 2–3 weeks after the ICIs was first administered and then decreased with the drug continuous administration^40^. Second, the mechanism behind lymphocytosis influencing TIL and neoantigen levels is still ambiguous. Lastly, we could not suggest a clinically useful cutoff for lymphocytosis because we used quartile analysis. Despite these limitations, this is the first study to determine whether PLC, not NLR, could be used as a simple surrogate marker. Compared to the current response evaluation happens after 2 or 3 cycles of ICIs (6–12 weeks) in general, using PLC can greatly shorten the period needed for response assessment to only about 4 weeks after ICIs. Therefore, the effectiveness of ICI based treatment in patients with NSCLC can be determined soon after initiation of therapy.

In conclusion, PFS was significantly longer with elevated pre and post-treatment PLCs in patients with advanced NSCLC treated with ICIs. Lymphocytosis pre- and post-treatment was not associated with irAEs. However, further studies are necessary to support these findings.

## Methods

### Study design and participants

We conducted a retrospective study from April 1, 2016, to March 31, 2019. Enrolled patients were aged ≥ 18 years, diagnosed with histologically proven NSCLC, received ICI-based (pembrolizumab, nivolumab, atezolizumab, or durvalumab) treatment, and underwent PD-L1 IHC at Seoul National University Hospital (SNUH). The study was approved by the Institutional Review Board (IRB no: H-1902-063-1010) of SNUH. Informed consent was waived due to the retrospective chart review study. The study was conducted in accordance with the principles of the Declaration of Helsinki. The patients were treated with pembrolizumab at a dose of 200 mg intravenously once every 3 weeks, nivolumab at a dose of 3 mg/kg of body weight once every 2 weeks, or atezolizumab at a dose of 1200 mg intravenously once every 3 weeks until disease progression.

### Variables

All patient records were retrospectively reviewed. Demographic variables (age at the first prescription of ICIs, sex, Eastern Cooperative Oncology Group (ECOG) status, and smoking status), tumor histology, anti-PD-L1 agents used, lines of treatment, and PD-L1 expression levels were noted^41^. The overall tumor response, including, complete, partial, stable, pseudo-progression, or progression, was defined as per the Immune Response Evaluation Criteria in Solid Tumors criteria^42^. Data on the objective response rate (ORR)—the proportion of patients who exhibited a partial or complete response to therapy—and PLC detected in the complete blood count test, before and after immunotherapy, were collected. Pre- and post-treatment PLC included the values measured within 1 week before ICIs initiation and at 3–4 weeks after initial ICIs administration, respectively. We also noted immune-related adverse events (irAEs); adverse events (AEs) were graded according to the National Cancer Institute Common Terminology Criteria for Adverse Events, version 4.0, and grades 3–5 of irAEs were defined as serious AEs.

### Analysis

The primary endpoint was progression-free survival (PFS) with immunotherapy, according to PLC. The secondary endpoints were overall survival (OS), ORR of ICIs, and AEs. Categorical variables were compared using the chi-square test or Fisher’s exact test, and continuous variables were compared using an independent unpaired t-test or Mann–Whitney test if variables were nonparametric. PLC was presented as medians with interquartile ranges. The quartile groups of PLC was compared using the Kruskal–Wallis statistical test. A multivariate Cox proportional hazard model was used to determine the differences between PFS and OS between the quartiles of PLC. We assumed that there would be a correlation between line of ICIs and other parameters, especially age. Therefore, a-priori Pearson’s correlation analysis was conducted to identify and exclude confounder (Supplementary Table 2). The line of ICIs had statistically significant negative correlation with the age (the correlation coefficient (r) =  − 0.13, *p* = 0.04), so we excluded the line of ICIs in the subsequent multivariate analysis. We adjusted the values for age, sex, smoking status, ECOG status, histology, epidermal growth factor receptor (EGFR) mutation status, and PD-L1 expression. The log-rank test was used to derive the trend in survival across the quartile groups of PLC. All statistical analyses were performed using Stata 13.0 (StataCorp, College Station, TX, USA), and a *p*-value of < 0.05 was considered statistically significant.

## Supplementary Information

Supplementary Tables.

Supplementary Figures.
