# Supplementary material for: Peripheral lymphocyte count as a surrogate marker of immune checkpoint inhibitor therapy outcomes in patients with non-small-cell lung cancer
Source: Sci Rep. 2022 Jan 12;12:626. doi: 10.1038/s41598-021-04630-9 (PMC8755768; doi:10.1038/s41598-021-04630-9)
Supplement: Supplementary file 1 — Supplementary Tables. [file 41598_2021_4630_MOESM1_ESM.docx]

Table S1. Multivariate analysis (Cox regression) of progression free survival.

|  | **aHR^*^** | **95% CI** | | **P value** |
| --- | --- | --- | --- | --- |
|  |  | Lower | Upper |  |
| Age^†^ | 0.96 | 0.94 | 0.98 | <0.001 |
| Sex(female) | 0.95 | 0.58 | 1.60 | 0.86 |
| Smoking (ref never) | 0.49 | 0.29 | 0.86 | 0.01 |
| ECOG (ref 0) |  |  |  |  |
| 1 | 2.08 | 1.12 | 3.84 | 0.02 |
| ≥2 | 3.60 | 1.83 | 7.06 | <0.001 |
| Histology (ref ADC) |  |  |  |  |
| Sqcc | 1.28 | 0.66 | 2.50 | 0.47 |
| NSCLC | 1.59 | 0.94 | 2.71 | 0.08 |
| EGFR mutation | 2.55 | 1.42 | 4.56 | 0.002 |
| PDL-1 expression | 0.52 | 0.32 | 0.84 | 0.006 |
| Pretreatment PLC | 0.99 | 0.99 | 1.00 | 0.007 |

*adjusted hazard ratio †age at first ICIs prescription

Table S2. Treatment related adverse events according to pretreatment PLC quartile groups.

| **Pretreatment PLC** | **Quart 1 (n=58)** | **Quart2**  **(n=58)** | **Quart3**  **(n=58)** | **Quart4**  **(n=57)** | **P** |
| --- | --- | --- | --- | --- | --- |
| Adverse event (n=61) | 11 (19.0%) | 15(18.0%) | 20 (32.8%) | 19 (31.2%) | 0.103 |
| Pneumonitis | 4 (30.7%) | 4 (36.4%) | 2 (10.0%) | 6 (31.6%) | 0.723 |
| Skin lesion | 2 (18.2%) | 0 (0.0%) | 4 (25.5%) | 4 (15.8%) |  |
| Hepatitis | 2 (9.1%) | 3 (27.3%) | 1 (5.0%) | 1 (5.3%) |  |
| TFT abnormality | 0 (0.0%) | 1 (9.1%) | 3 (15.0%) | 2 (10.5%) |  |
| Arthritis | 0 (0.0%) | 0 (0.0%) | 1 (5.0%) | 2 (10.5%) |  |
| Fever | 1 (9.1%) | 0 (0.0%) | 1 (5.0%) | 1 (5.3%) |  |
| Anorexia | 1 (0.0%) | 1 (9.1%) | 1 (5.0%) | 1 (5.3%) |  |
| Others | 3 (27.3%) | 2 (18.2%) | 7 (30.0%) | 4 (15.8%) |  |
| Serious adverse event | 5 (8.5%) | 3 (5.1%) | 7 (11.9%) | 6 (10.2%) | 0.670 |

Table S3. Correlation of study parameters by Pearson’s correlation analysis.

| Study parameters | Line of ICIs | Age | Sex | Smoking | ECOG | Histology | EGFR | PD-L1 expression | Pre-treatment PLC | Post-treatment PLC |
| --- | --- | --- | --- | --- | --- | --- | --- | --- | --- | --- |
| Line of ICIs | 1 | -0.130* | 0.109* | 0.042 | -0.032 | -0.148^*^ | 0.179^*^ | -0.133^*^ | 0.009 | 0.033 |
| Age | -0.130* | 1 | -0.110 | 0.113 | 0.170^*^ | 0.137^*^ | -0.116 | 0.152^*^ | 0.085 | 0.018 |
| Sex | 0.109* | -0.110 | 1 | -0.237^*^ | -0.050 | -0.159^*^ | 0.322^*^ | -0.068 | -0.075 | -0.059 |
| Smoking | 0.042 | 0.113 | -0.237^*^ | 1 | 0.225^*^ | 0.001 | -0.057 | 0.184^*^ | 0.080 | 0.104 |
| ECOG | -0.032 | 0.170^*^ | -0.050 | 0.225^*^ | 1 | 0.071 | -0.069 | 0.007 | -0.004 | -0.010 |
| Histology | -0.148^*^ | 0.137^*^ | -0.159^*^ | 0.001 | 0.071 | 1 | -0.155^*^ | -0.021 | 0.072 | 0.274 |
| EGFR | 0.179^*^ | -0.116 | 0.322^*^ | -0.057 | -0.069 | -0.155^*^ | 1 | -0.002 | -0.013 | 0.026 |
| PD-L1 expression | -0.133* | 0.152^*^ | -0.068 | 0.184^*^ | 0.006 | -0.021 | -0.002 | 1 | -0.021 | -0.017 |
| Pre-treatment PLC | 0.009 | 0.085 | -0.075 | 0.080 | -0.004 | 0.072 | -0.013 | -0.021 | 1 | 0.624^*^ |
| Post-treatment PLC | 0.033 | 0.018 | -0.059 | 0.104 | -0.010 | 0.274 | 0.026 | -0.017 | 0.624^*^ | 1 |
