# Supplementary material for: Peripheral lymphocyte count as a surrogate marker of immune checkpoint inhibitor therapy outcomes in patients with non-small-cell lung cancer
Source: Sci Rep. 2022 Jan 12;12:626. doi: 10.1038/s41598-021-04630-9 (PMC8755768; doi:10.1038/s41598-021-04630-9)
Supplement: Supplementary file 2 — Supplementary Figures. [file 41598_2021_4630_MOESM2_ESM.docx]

Supplementary Figure S1. Kaplan-Meier curves showing progression-free survival (PFS) stratified by quartiles of pre-treatment PLC


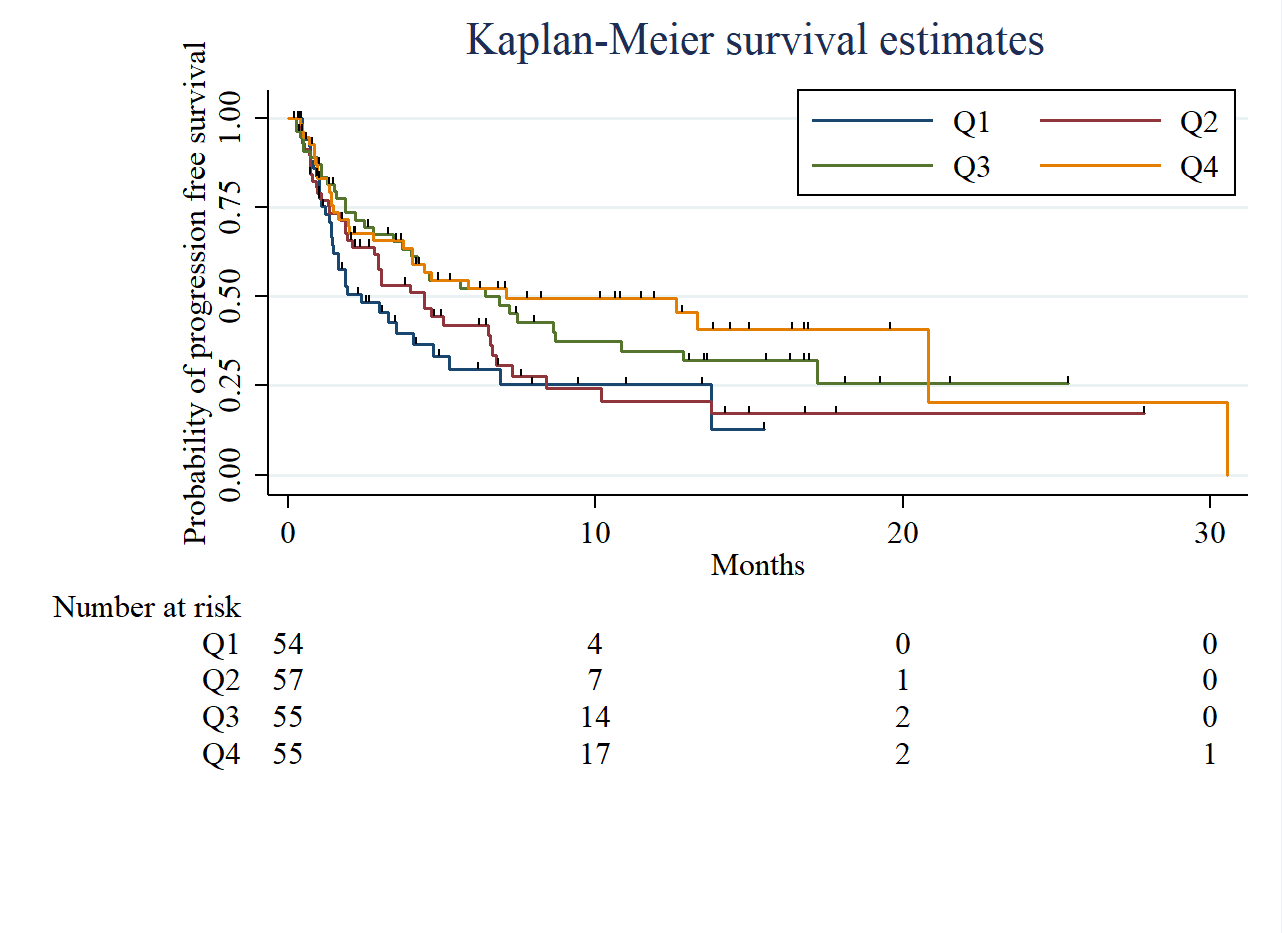


Supplementary Figure S2. A) Kaplan-Meier curves showing overall survival (OS) stratified by quartiles of pre-treatment PLC. B) Kaplan-Meier curves showing overall survival (OS) stratified by quartiles of post-treatment PLC.


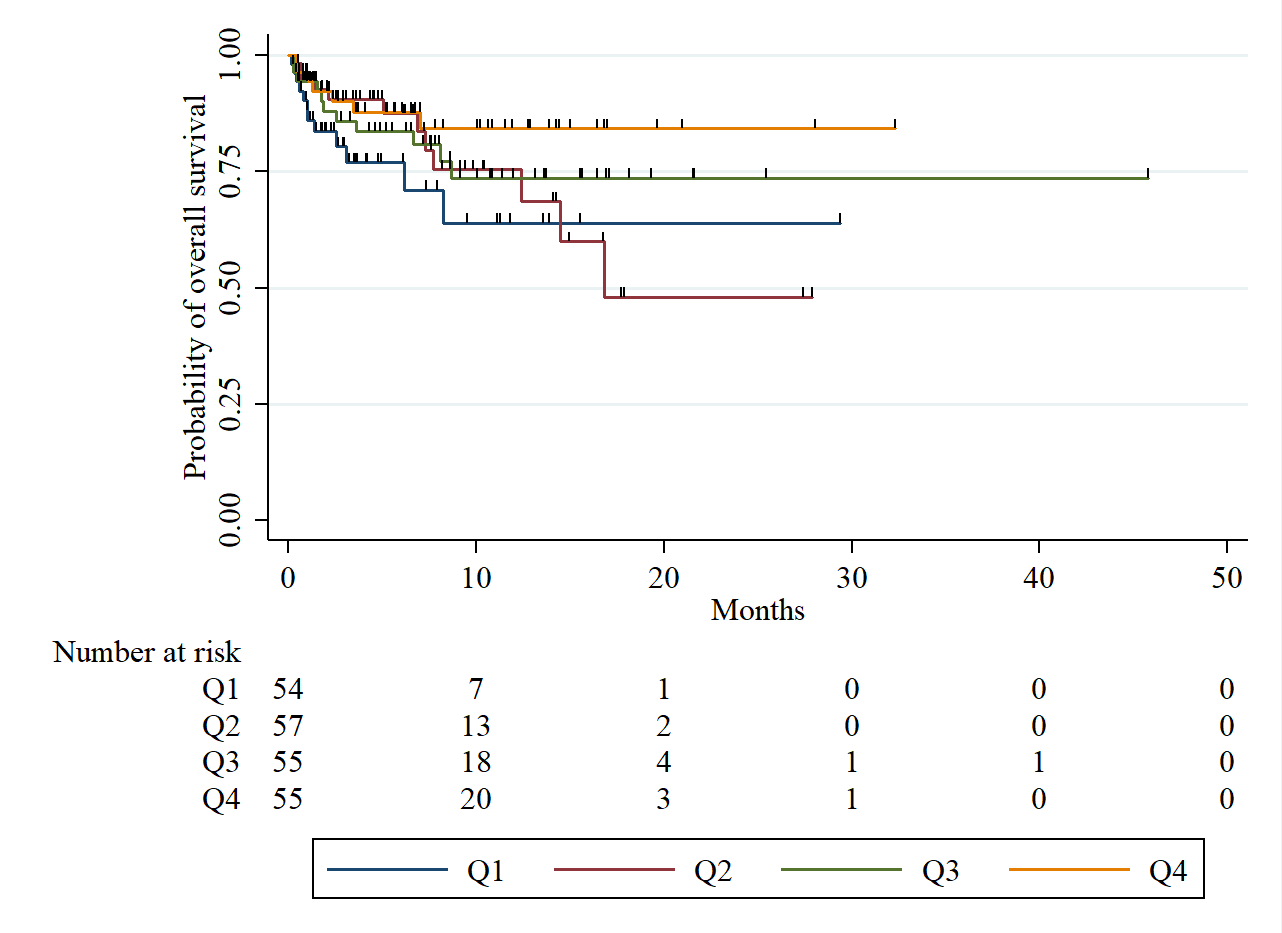

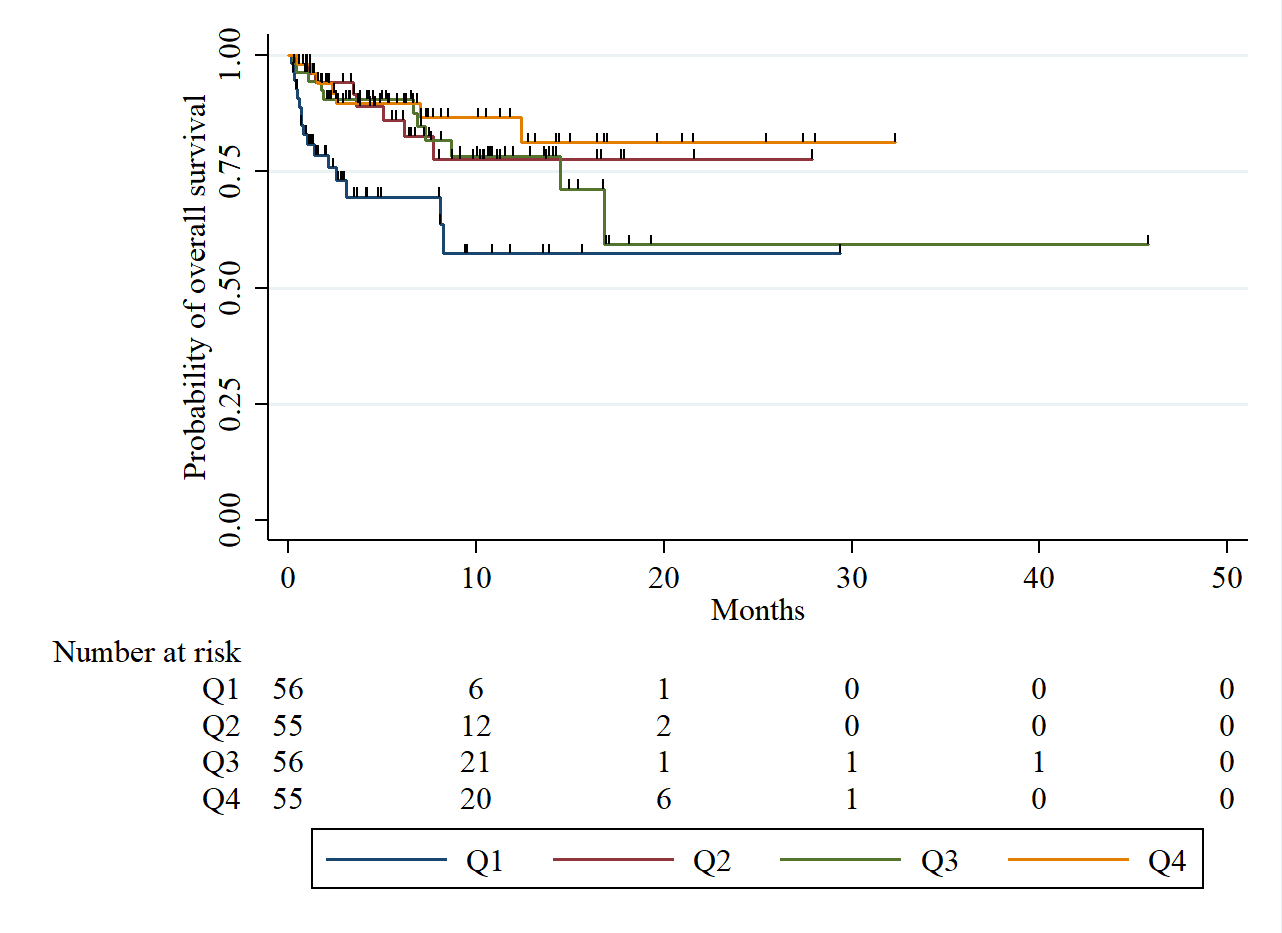


**B)**

**A)**
